# Application Performance Evaluation of an Ionic-Liquid-Enhanced Composite Drag Reducer with Pour-Point-Depressing Functionality

**DOI:** 10.3390/molecules31203668

**Published:** 2026-10-14

**Authors:** Penggang Zhang, Mingji Huang, Yufa Deng, Hongzhou Chao, Donglei Yang, Hancheng Li, Chi Yi, Junkai Zhu

**Affiliations:** Tarim Oilfield Company, PetroChina, Korla 841000, China; zhangpenggang-tlm@petrochina.com.cn (P.Z.); huangmingji-tlm@petrochina.com.cn (M.H.); dengyufa-tlm@petrochina.com.cn (Y.D.); chaohongzhou-tlm@petrochina.com.cn (H.C.); lihancheng-tlm@petrochina.com.cn (H.L.); yichi-tlm@petrochina.com.cn (C.Y.); zhujunkai-tlm@petrochina.com.cn (J.Z.)

**Keywords:** composite drag reducer, ionic liquid, high-wax crude oil, drag reduction rate, rheological properties

## Abstract

To address the critical problems of wax crystallization and deposition, high viscosity and friction, and pipeline gelling risk during the pipeline transportation of high-wax crude oil, a PAO/IL/JN-3 ternary composite drag reducer was constructed via physical blending, using poly(α-olefin) (PAO) as the drag-reducing main agent, JN-3 as the pour point depressant component, and ionic liquid as the functional regulator, and its application performance was systematically evaluated. Proportion optimization showed that the optimal mass ratio of PAO to JN-3 was 1:2, IL-C14 provided the best enhancement, and the optimal mass fraction of IL was 30 wt%. Rheological tests showed that at a concentration of 40 mg/kg, the storage modulus G′ was approximately 82% lower and the loss modulus G″ was approximately 78% lower than those of the PAO-only system. Wax deposition evaluation showed that for the model oil containing 30 wt% paraffin, the reduction rate of the wax deposition amount reached 38% at 40 mg/kg. In the flow loop tests, a drag reduction rate of 50.5% was achieved at 40 mg/kg, and in the field pipeline test, the pressure drop decreased from 2.15 MPa to 1.15 MPa, corresponding to an average drag reduction rate of 46.5%. Overall, the ionic liquid reconciles the functional conflict between the drag-reducing and pour-point-depressing components, and the PAO/IL/JN-3 ternary composite drag reducer integrates drag reduction with pour-point-depressing functionality inferred from rheological, wax deposition, and crystal morphology evidence, providing a theoretical basis for the pipeline transportation of high-wax crude oil.

## 1. Introduction

The crude oil produced in the Tarim Oilfield, China, is mostly high-wax crude oil characterized by a high pour point, high viscosity, and poor low-temperature flowability [1,2]. During transportation from gathering stations to long-distance pipelines, the crude oil continuously loses heat to the environment; wax molecules gradually precipitate and form crystal networks of a certain strength, while some wax crystals become enriched and deposit near the pipe wall, resulting in a reduced effective flow cross-section, increased flow resistance, and decreased transportation capacity, and in severe cases this issue may even trigger pipeline gelling accidents [3,4,5]. The pressure and dissolved gas in the pipeline also significantly affect the wax appearance temperature and wax deposition behavior of crude oil [6]. At present, heated transportation is the main engineering measure used to maintain the fluidity of crude oil. However, it suffers from high energy consumption, insufficient operational flexibility, and a high restart risk after shutdown. Therefore, the development of chemical additives that can simultaneously improve turbulent friction and low-temperature flowability is of great significance for the safe and economical transportation of high-wax crude oil.

The addition of drag reducing agents (DRAs) is an important technical means of reducing pipeline friction and improving pipeline transportation capacity. Since Toms discovered the turbulent drag reduction phenomenon of polymer solutions [7], polymer-based DRAs have gradually become the dominant type of crude oil pipeline DRA owing to their high drag reduction rate and low required concentration. At present, oil-soluble DRAs are mainly represented by polyisobutylene (PIB), poly(α-olefin) (PAO), and methacrylate-type polymers, whose core functions are generally associated with the extension and orientation of polymer chains in turbulence and their regulation of the near-wall turbulent structures [8,9,10,11,12,13,14]. In recent years, research interest has gradually shifted from simply increasing the drag reduction rate toward improving adaptability to complex operating conditions, mainly reflected in the following approaches: enhancing the drag reduction capability by increasing the polymer molecular weight and optimizing the chain architecture; constructing composite structures with inorganic components such as nano-SiO_2_ and montmorillonite to improve shear stability; and extending the temperature resistance, medium tolerance, and rapid-dissolution performance through hydrophobically associating structures, functional copolymerization, and bio-based materials [15,16,17].

However, existing oil-soluble polymer DRAs still exhibit prominent limitations. First, the drag reduction performance is highly sensitive to the polymer molecular weight and chain integrity; the polymer chains are prone to mechanical degradation in local high-shear and extensional flow regions such as pumps, valves, and elbows, leading to a decrease in molecular weight and a decay of the drag reduction rate. Recent circulating flow tests on PIB showed that its drag reduction rate decreased markedly after repeated exposure to high shear [18]. Second, the dissolution and dispersion of high-molecular-weight DRAs and their compatibility with the crude oil components directly affect practical applications; agglomeration, precipitation, or uneven dispersion reduces the effective utilization of the additive and may even increase transportation risks [19]. Third, traditional DRAs mainly regulate the turbulent friction and have limited ability to directly control wax nucleation, growth, and three-dimensional network formation; therefore, under the low-temperature transportation conditions of high-wax crude oil, the use of a DRA alone can hardly fundamentally improve the low-temperature fluidity of the crude oil, nor can it effectively suppress the abrupt increase in low-temperature viscosity and the wax deposition [20,21,22,23]. This has driven research on pipeline DRAs gradually from “single drag reduction” toward the “synergy of drag reduction and flow assurance”.

For the low-temperature transportation of high-wax crude oil, pour point depressants (PPDs) mainly regulate the wax nucleation and growth processes through adsorption, co-crystallization, steric hindrance, and dispersion, altering the size, morphology, and aggregation mode of the wax crystals and thereby weakening the formation of the wax crystal network [24,25]. Common PPD components include EVA and its modified polymers, acrylate polymers, poly(α-olefin) derivatives, and the nanocomposite PPDs developed in recent years; among them, the long-chain nonpolar segments can co-crystallize or adsorb with the wax molecules, whereas the polar segments hinder further regular growth of the wax crystals through steric hindrance and interfacial interactions [26,27,28,29]. In recent years, PPD research has gradually evolved from single polymers toward polymer blends, nano-hybridization, and structural functionalization, in order to simultaneously improve the depression magnitude, aging resistance, and adaptability to complex crude oils [30,31,32]. However, PPDs have a limited ability to regulate the turbulent structures and can hardly replace highly efficient DRAs alone to achieve significant drag reduction performance.

To integrate the drag reduction and pour point depression functions, existing studies mainly follow two technical routes: chemical copolymerization and physical blending. Chemical copolymerization, grafting, or block strategies introduce the structural units with both drag-reducing and wax-regulating functions into the same polymer chain, which can reduce the compatibility problems arising from the independent migration of multiple components and is expected to achieve functional synergy; however, the molecular structure design and polymerization processes are complex, usually requiring trade-offs among the molecular weight, branching degree, polarity, and solubility, which leaves little room for performance adjustment and makes it difficult to adapt to varying crude oil properties and transportation conditions. In contrast, physical blending directly mixes polymer DRAs such as PAO and PIB with PPDs such as EVA and acrylate polymers; this method is simple to prepare and flexible in component selection, allowing the formulation to be adjusted at any time according to the crude oil properties and field conditions, which facilitates field application [33]. In addition, constructing composite systems with inorganic components such as nano-SiO_2_ can also balance the shear stability and the wax inhibition capability. Nevertheless, the performance of a drag reduction–pour point depression blended system is not a simple superposition of its components: on the one hand, the DRA requires the polymer to maintain a good dissolution state and an extended chain conformation, whereas the PPD needs to preferentially participate in the wax crystal formation and function at the wax crystal/oil interface; the two components differ in both the target and the scale of action, and they are thus prone to competitive adsorption and spatial competition. On the other hand, the enhanced polarity of the PPD component, although favorable for interacting with the wax crystals, resins, and asphaltenes, may weaken the dissolution and dispersion of nonpolar polymers such as PAO in the crude oil and even alter the effective conformation of the polymer chains [34]. Therefore, establishing a stable and compatible “drag reduction-pour point depression” synergistic structure within a single system is the key to the further development of composite pipeline DRAs. This provides a new approach for introducing structurally designable functional components such as ionic liquids into blended systems to stabilize the compatibility relationships among multiple components.

Ionic liquids (ILs) feature designable cation-anion structures, tunable polarity and hydrophobicity, good interfacial activity, and a wide liquid temperature range, providing a new material design space for solving the interfacial compatibility and wax regulation problems in the blended systems. Studies have shown that ionic liquids can directly exhibit pour point depression and flow modification effects and significantly improve the low-temperature flow performance of high-wax crude oils; ionic liquids represented by 1-octyl-3-methylimidazolium chloride have been applied to the pour point depression of high-wax crude oil, showing good performance in reducing the pour point and apparent viscosity and in inhibiting wax deposition [35]. Recent studies further indicate that functionalized ionic liquids with pour point depression and flow improvement effects can be obtained by tuning the alkyl chain of the cation and the anion structure. In terms of mechanism, ionic liquids can interfere with the wax nucleation, growth, and aggregation through the interactions between their hydrophobic segments and the long-chain alkanes/wax molecules, thereby altering the wax crystal morphology and spatial network; on the other hand, their ionic nature and tunable polarity are expected to improve the interfacial compatibility and dispersion among the DRAs, PPDs of different polarities, and the polar components of the crude oil, thus alleviating the agglomeration, precipitation, and functional competition in multi-component blended systems. For polymer DRAs, ionic liquids may also regulate the dispersion and effective conformation of the polymer chains by altering the local solvation environment and the polymer–oil interactions, thereby providing molecular-level regulation for the synergy between the drag reduction and pour point depression. It should be noted that the effect of an ionic liquid is strongly structure-dependent; the improvement depends on the cation, the anion, the alkyl chain length, and their matching with the PAO/PPD and the crude oil components, so ionic liquids are more suitable to serve as a “functional regulator” rather than a simple third PPD. Compared with previous studies in which imidazolium-based ionic liquids with different anions were used alone as pour point depressants or flow improvers [36], the present work introduces the ionic liquid as a functional regulator into a DRA + PPD blended system to reconcile the functional conflict between the two components, which constitutes the novelty of this study. Meanwhile, the potential limitations of ionic liquids, including relatively high cost, possible toxicity and environmental impact, and difficult recyclability, should be carefully considered for large-scale field applications.

Based on the above understanding, in this study an ionic liquid was further introduced into the composite system composed of the PAO drag-reducing main agent and the JN-3 pour point depressant to construct a pour-point-depressing multi-component composite drag reducer, expanding the research system from the traditional binary “DRA + PPD” physical blend to a ternary “drag reduction-pour point depression-interfacial regulation” synergistic system. The compatibility stability, wax deposition characteristics, wax crystal morphology, rheological properties, drag reduction rate, and wax deposition inhibition performance of the system after the addition of the ionic liquid were investigated, the influence of the ionic liquid on the synergy between PAO and JN-3 and its possible mechanisms were analyzed, and the practical applicability was evaluated through pipeline simulation tests. This study aims to elucidate the synergistic law between the drag reduction and pour point depression functions in multi-component composite systems, as well as to provide a theoretical basis and technical support for the transition of high-wax crude oil pipelines from sole heated transportation to chemical regulation and low-energy-consumption transportation.

## 2. Results and Discussion

### 2.1. Characterization of the Composite Drag Reducer

#### 2.1.1. FT-IR Analysis

Figure 1 shows the FT-IR spectrum of the PAO/IL/JN-3 composite drag reducer. The strong characteristic peaks at 2920 cm^−1^ and 2850 cm^−1^ are attributed to the asymmetric and symmetric stretching vibrations of the methylene groups (-CH_2_-) in the PAO molecules, respectively; the peak at 2955 cm^−1^ is assigned to the asymmetric stretching vibration of the terminal methyl groups in the PAO molecules; the absorption peak at 1465 cm^−1^ corresponds to the scissoring vibration of the methylene groups in the PAO and pour point depressant molecules; the peak at 1380 cm^−1^ is attributed to the symmetric deformation (bending) vibration of the methyl groups in the two polymer chains; and the peak at 720 cm^−1^ is assigned to the in-plane rocking vibration of (CH_2_)_n_ (n ≥ 4) in the long side-chain structure of the PAO molecules, which constitutes direct evidence for the presence of long-chain alkyl groups.

The characteristic peak at 1740 cm^−1^ belongs to the carbonyl stretching vibration of the ester groups in the pour point depressant molecules; the peaks at 1260 cm^−1^ and 1170 cm^−1^ are attributed to the asymmetric and symmetric stretching vibrations of the C-O-C bonds in the ester groups of the pour point depressant molecules, respectively. The presence of these three ester characteristic peaks confirms that the pour point depressant has been successfully introduced into the composite system.

The characteristic peak at 1570 cm^−1^ is assigned to the C=N stretching vibration of the imidazole ring of the ionic liquid molecules, which is the most diagnostic absorption peak of the ionic liquid; the weak characteristic peak at 3150 cm^−1^ belongs to the stretching vibration of the C-H bonds of the imidazole ring; the peaks at 3050–3100 cm^−1^ are attributed to the stretching vibration of the C-H bonds at the 4- and 5-positions of the imidazole ring (=C-H); and the peak at 850 cm^−1^ corresponds to the out-of-plane bending vibration of the C-H bonds on the imidazole ring.

The characteristic peaks of the individual components in the ternary system do not overlap with each other; no new characteristic absorption peaks were observed, and no significant shifts in the existing characteristic peaks were found. These results indicate that no chemical reaction occurred among the molecules of the ternary system; the three components retain their respective independent chemical structures in the composite drag reducer, forming a physical mixture with good compatibility.

#### 2.1.2. DSC Analysis

Figure 2 shows the DSC curves of the ternary composite drag reducer. As can be seen, a broad exothermic peak appears in the low-temperature region during the cooling process, corresponding to the ordered arrangement and crystallization of the long-chain alkyl groups at low temperature; no distinct melting peak is observed within the measured temperature range (−40 °C to 80 °C).

The composite drag reducer exhibits no endothermic or exothermic behavior within the range of −40 °C to 80 °C; no glass transition was observed on the heating curve, and no crystallization peak appeared in this temperature range during the cooling process. According to polymer thermodynamic theory, the glass transition temperature reflects the segmental mobility of the polymer chains, whereas the crystallization peak is related to the crystallization behavior of the polymer. It should be noted that the glass transition temperature of PAO-based systems may be lower than −40 °C, so the absence of a Tg signal cannot be simply ascribed to restricted segmental mobility. Nevertheless, the absence of any glass transition, crystallization, or melting event within −40 °C to 80 °C indicates that the composite drag reducer undergoes no phase or aggregation-state change in the temperature window relevant to conventional crude oil pipeline transportation, which supports its performance stability in practical application.

#### 2.1.3. TGA Analysis

Figure 3 shows the TGA curve of the ternary composite drag reducer. The mass of the composite drag reducer remains essentially stable from room temperature to 340 °C, with a weight loss of only 7.15%. When the temperature exceeds 340 °C, the sample begins to lose weight rapidly; within the range of 340–460 °C, the mass drops sharply from 92.85% to 0%, which is caused by the thermal decomposition of the PAO and JN-3 molecules. The TGA results indicate that the composite drag reducer possesses good thermal stability below 340 °C and can satisfy the application requirements under the conventional crude oil pipeline transportation temperatures (<80 °C).

### 2.2. Proportion Optimization of the Ternary Composite Drag Reducer

#### 2.2.1. Optimization of the PAO/JN-3 Mass Ratio

At room temperature, the DRA systems were injected into the flow loop apparatus at a concentration of 40 mg/kg, and the drag reduction rates were measured and calculated. As shown in Figure 4, the drag reduction rate of PAO used alone is only 30.3%. This is because in the model oil system, once the oil temperature approaches the wax appearance point, the precipitated paraffin crystals interlock with one another to form a three-dimensional network, which not only significantly increases the apparent viscosity and yield stress of the system, but also imposes steric hindrance and mechanical entanglement on the extension and orientation of the ultra-high-molecular-weight flexible chains, limiting the ability of the PAO molecules to respond to the turbulent bursts within the turbulent buffer layer and thus preventing its drag reduction potential from being fully released. After the introduction of the JN-3 pour point depressant, the drag reduction rate increases from 30.3% to 36.1% with an increasing blending ratio, with the most significant increase occurring in the range of 1:1.5–1:2. This is attributed to the co-crystallization/adsorption interaction between the long alkyl side chains in the JN-3 molecular structure and the paraffin crystals, which can distort the paraffin crystal growth habit and destroy the continuity of the three-dimensional paraffin crystal network, refining and dispersing the paraffin crystals, thereby reducing the viscoelastic resistance of the continuous phase and providing a favorable rheological environment for the extension and orientation of the PAO chains in the shear field. However, when the ratio exceeds 1:2, the drag reduction rate reaches a plateau, and a further increase in the JN-3 content provides no significant benefit. The underlying reason lies in the inherent conflict in the molecular design of the two additives: the drag reduction function requires molecules with the maximum molecular weight and maximum chain flexibility needed to strengthen their elastic response in the turbulent buffer layer, whereas the paraffin crystal modification function requires molecules carrying long alkyl side chains that match the paraffin segments and polar groups that provide steric hindrance, which inevitably comes at the cost of the chain flexibility and the upper limit of the molecular weight. Therefore, PAO/JN-3 (1:2) was selected as the optimal ratio.

#### 2.2.2. Effect of the Ionic Liquid Structure on the Drag Reduction Rate

Figure 5 shows the variations in the drag reduction rate when different ionic liquids (10 wt%) were added to the PAO/JN-3 (1:2) binary system. After the introduction of the alkyl-chain ionic liquids, the drag reduction rates of the ternary systems are significantly higher than that of the PAO/JN-3 binary system; among them, the PAO/IL-C14/JN-3 system achieves the highest drag reduction rate, and the PAO/IL-C12/JN-3 and PAO/IL-C16/JN-3 systems reach 43.2% and 42.0%, respectively, whereas the drag reduction rate of the benzene-ring-containing PAO/IL-Ben/JN-3 system is only 35.8%, which is close to that of the binary system. These results indicate that the introduction of ionic liquids can effectively reconcile the molecular design conflict between the PAO and the JN-3: the PAO possesses an ultra-high molecular weight and maximum chain flexibility, whereas the JN-3 provides polar groups that impart steric hindrance. The ionic liquid has a typical amphiphilic structure; its polar imidazolium group can interact with the polar functional groups of JN-3 through electrostatic, hydrogen-bonding, or π–π interactions, while its nonpolar long alkyl tail can undergo hydrophobic association with the PAO backbone and the paraffin crystals, It is therefore hypothesized that the ionic liquid may act as a “molecular bridge” between PAO and JN-3, enabling the spatial decoupling and synergistic action of the two functional components within the same system. The alkyl chain length has a significant influence on the synergistic effect: the C12 chain is too short to provide sufficient hydrophobic bridging capability; the C16 chain is too long and tends to induce the self-aggregation of the IL, forming a local viscoelastic resistance and shielding the active sites of JN-3; the C14 chain is of an intermediate length, which can effectively match the paraffin crystal lattice while forming a moderate association with the PAO backbone, and therefore exhibits optimal drag reduction enhancement. In contrast, the benzene-ring-substituted ionic liquid fails to improve the drag reduction rate, because the rigid planar structure of the aromatic ring can hardly co-crystallize with the orthorhombic crystal system of paraffin, and the benzene ring is poorly compatible with the nonpolar PAO backbone, so it cannot exert a bridging effect. In summary, under identical flow-loop conditions, the drag reduction rate increases from 30.3% for PAO alone to 36.1% for the PAO/JN-3 binary system and further to 44.4–50.3% for the ternary formulations, an increment of about 14 percentage points over the binary system, which supports a synergistic rather than merely additive effect of the three components.

Figure 6 shows the effect of the mass fraction of the ionic liquid (IL-C14) in the PAO/IL/JN-3 ternary composite drag reducer on the drag reduction rate. When the IL mass fraction increases from 10 wt% to 30 wt%, the drag reduction rate rises rapidly from 44.4% to 50.3%; it maintains a plateau maximum within the range of 30–40 wt%. When the mass fraction is further increased to 50 wt%, the drag reduction rate decreases, and at 60 wt%, it declines even more significantly. When the IL content is too low, the interfacial bridging density is insufficient to fully reconcile the aforementioned conflict, resulting in a relatively low drag reduction rate; when the IL content reaches 30 wt%, the PAO/IL/JN-3 ternary supramolecular network tends to be complete, the synergistic effect reaches saturation, and the drag reduction rate maintains its maximum value. However, when the IL content exceeds 40 wt%, the effective concentrations of PAO and JN-3 are diluted, and the excessive IL self-aggregates in the oil phase to form local viscoelastic structures, which not only increases the flow resistance of the continuous phase but also restricts the free extension of the PAO molecular chains, leading to a significant decrease in the drag reduction rate. Since the drag reduction rates at 30 wt% and 40 wt% are basically consistent, 30 wt% is selected as the optimal IL proportion according to the minimum effective dosage principle, which reduces the formulation cost and avoids the potential adverse effects of excessive ionic liquid on the oil properties.

### 2.3. Effect of the Composite Drag Reducer on the Rheological Properties of the Model Oil

As shown in Figure 7, the storage modulus G’ of the diesel remains close to zero over the entire test temperature range (25–40 °C), indicating that the diesel matrix is a typical viscous fluid that does not form an elastic network structure. After the addition of 20 wt% paraffin, the system exhibits a distinct peak near 26 °C, corresponding to the massive deposition of paraffin and the formation of a continuous three-dimensional paraffin crystal skeleton through van der Waals forces and mechanical entanglement. Thereafter, the paraffin crystals gradually melt with increasing temperature, and G’ decreases slowly, approaching zero at approximately 38 °C.

The addition of 40 mg/kg PAO alone significantly increases G’, which is 3.3 times that of the paraffin system. This phenomenon indicates that the long PAO chains may participate in the co-crystallization of the paraffin crystals or adsorb on the surface of the wax nuclei, promoting the formation of more continuous paraffin crystal aggregates and thereby strengthening the elastic stiffness of the gel network; using PAO alone not only fails to improve the low-temperature fluidity of the model oil, but also aggravates the formation of the elastic structure. In contrast, the G’ curve of the composite drag reducer (40 mg/kg) shifts markedly downward: at 30 °C, G’ is approximately 82% lower than that of the PAO-only system, and at 35 °C, the system already approaches fluid behavior. These data indicate that the ternary composite system effectively weakens the strength of the three-dimensional paraffin gel network by increasing the number of nucleation sites, refining the paraffin crystal size, and inhibiting the interlocking of the needle-like paraffin crystals, while offsetting the enhancing effect of PAO alone on G’. Overall, the G’ of the composite drag reducer in the critical temperature range of 26–35 °C is 68–82% lower than that of the PAO-only system, demonstrating the synergistic advantages of drag reduction and wax crystal modification and significantly improving the low-temperature rheological properties of the high-paraffin crude oil. Since the gelation and pour point behavior of waxy oils is governed by the strength of the wax crystal network, the marked weakening of G′ and G″ indicates an improved low-temperature flowability, consistent with the generally accepted correlation between the gel structure and the pour point of waxy crude oils [37].

As shown in Figure 8, the loss modulus G″ of the diesel remains close to zero over the entire range of 25–40 °C, indicating that the diesel matrix contains almost no viscous dissipation structure and exhibits typical fluid characteristics. After the addition of 20 wt% paraffin, the system shows a distinct peak near 26 °C, corresponding to the energy dissipation dominated by viscous flow during the paraffin deposition and the formation of the three-dimensional paraffin crystal network; as the temperature increases, the paraffin crystals gradually melt and G″ decreases continuously until approaching zero. The addition of 40 mg/kg PAO alone significantly increases G″: the peak at 26 °C rises to approximately 7900 Pa, which is about 1.5 times that of the paraffin system, and G″ is still maintained at approximately 6400 Pa at 30 °C. This indicates that the presence of PAO alone likewise strengthens the viscous dissipation structure of the model oil, increasing the internal friction and flow resistance of the fluid at low temperature, which is consistent with the G’ results and further confirms that PAO alone is unfavorable for improving the low-temperature rheological properties of the model oil. In contrast, the G″ curve of the composite drag reducer (40 mg/kg) shifts markedly downward: at 30 °C, G″ is approximately 1400 Pa, which is about 78% lower than that of the PAO-only system, and at 35 °C, the system is already close to the fluid state. These results indicate that the ternary composite system effectively weakens the viscous dissipation structure of the system by refining the paraffin crystals and inhibiting their interlocking and offsets the enhancing effect of PAO alone on G″. Overall, in the critical temperature range of 26–35 °C, the composite drag reducer significantly reduces the viscous flow resistance while weakening the elastic wax crystal network, reflecting good drag reduction–wax modification synergy.

As shown in Figure 9, the torque M of the diesel remains close to zero throughout the test range, indicating that the diesel produces almost no structural resistance under rotational shear and exhibits typical fluid characteristics. After the addition of 20 wt% paraffin, the system shows a distinct peak near 26 °C, corresponding to the massive deposition of paraffin and the formation of the three-dimensional paraffin crystal network, which causes the system to exhibit significant structural resistance under rotational shear; as the temperature increases, the paraffin crystal network gradually melts and disintegrates, and M decreases continuously. The addition of 40 mg/kg PAO alone significantly increases the torque, indicating that the presence of PAO alone markedly enhances the structural strength and shear resistance of the model oil, which corroborates the G’ and G″ results and further demonstrates that the use of PAO alone deteriorates the low-temperature flow performance of the model oil. In contrast, the torque curve of the composite drag reducer (40 mg/kg) shifts markedly downward; its peak at 26 °C is not only far below that of the PAO-only system but also below that of the pure paraffin system. These results indicate that the ternary composite system effectively reduces the rotational shear resistance of the system by changing the paraffin crystallization morphology, refining the paraffin crystal size, and inhibiting the crosslinking of the paraffin crystal network, and it completely offsets the enhancing effect of PAO alone on the torque. Overall, the torque of the composite drag reducer within the test temperature range is 65–80% lower than that of the PAO-only system and is also significantly lower than that of the paraffin system, indicating that the composite system significantly reduces the structural resistance during the actual shear process while reducing the elastic and viscous moduli, exhibiting good drag reduction–wax modification synergy and contributing to the reduction in pumping energy consumption during the low-temperature transportation of high-paraffin crude oil.

### 2.4. Evaluation of the Wax Deposition Behavior

#### 2.4.1. Analysis of the Wax Deposition Amount

The reduction rate of the paraffin deposition amount is defined as the percentage decrease in the precipitated wax amount under the same conditions, which is suitable for quantifying the wax inhibition effect. As shown in Figure 10A, in the PAO/IL/JN-3 composite drag reducer system, the reduction rate of the paraffin deposition amount of each model oil increases with increasing composite drag reducer concentration and levels off at approximately 40 mg/kg; moreover, at the same concentration, the higher the paraffin content, the higher the reduction rate of the paraffin deposition amount. The wax inhibition effect is most significant for the model oil containing 30 wt% paraffin: the reduction rate rises rapidly to approximately 25% at 20 mg/kg and reaches 38% at 40 mg/kg. For the model oil containing 20 wt% paraffin, the reduction rate stabilizes at 25% at 40 and 50 mg/kg. For the model oil containing 15 wt% paraffin, the reduction rate increases relatively gently and stabilizes at approximately 15% at 40–50 mg/kg. For the model oils containing 10 wt% and 5 wt% paraffin, the reduction rates remain basically below 10%, indicating that a further increase in the PAO/IL/JN-3 concentration provides limited improvement in the wax inhibition effect at low paraffin contents.

Figure 10B shows the comparative trend of the PAO/IL/EVA composite system. Although this system shows a similar trend, the reduction rates of the paraffin deposition amount at the same concentration are all lower than those of the PAO/IL/JN-3 composite drag reducer, implying that the EVA system requires a higher concentration to achieve an effect comparable to that of PAO/IL/JN-3.

The reason for this result is that the core function of IL is to “bridge” the PAO and PPD components into an integrated synergistic assembly, thereby reconciling the inherent mismatch between the two in terms of molecular weight and chain flexibility. Moreover, owing to the strong ion–dipole and hydrogen-bonding interactions between JN-3 and the IL, the IL can effectively disperse the PPD into PAO, thereby delivering a markedly better performance than EVA.

#### 2.4.2. Analysis of the Wax Deposition Time

As shown in Figure 11, in the absence of any additive, obvious paraffin deposits occur in the model oil containing 30 wt% paraffin within 0.5 h, and 90% of the paraffin deposits within 2 h. This indicates that after 2 h, the paraffin deposition layer on the cold finger surface has already formed a relatively stable heat/mass transfer resistance, and the flux of the wax molecules that can migrate to the cold finger surface in the oil sample decreases significantly.

After the addition of 10 mg/kg of the composite drag reducer, the paraffin deposition amount decreases to a certain extent, but the effect is limited. At 0.5 h, the deposition amount is close to that of the blank group; at 2 h, the paraffin deposition amount is approximately 11.1% lower than that of the blank group; and, subsequently, the deposition rate remains relatively fast, and 93.3% of the paraffin has deposited by 10 h. This indicates that a concentration of 10 mg/kg has no obvious inhibitory effect on the initial stage of paraffin deposition and only delays the rapid growth stage of deposition to a certain extent and hardly changes the deposition habit of the paraffin crystals.

When the concentration of the composite drag reducer is increased to 40 mg/kg, the paraffin deposition amount decreases significantly. At 0.5 h, the deposition amount is approximately 17.6% lower than that of the blank group; after 6 h, it basically reaches a plateau, approximately 8.7% lower than that of the blank group. These results indicate that at a concentration of 40 mg/kg, the molecules of the ternary composite system can effectively encapsulate the paraffin crystal nuclei and inhibit the growth and coalescence of the paraffin crystals, while reducing the adhesion and deposition of the paraffin crystals on the cold finger surface by changing the paraffin crystal morphology (from needle-like to fine granular), thereby significantly reducing the paraffin deposition amount. A further increase in concentration to 50 mg/kg brings no obvious improvement in the inhibition effect. Judging from the overall data in Figure 10 and Figure 11, the comprehensive wax inhibition performance of the PAO/IL/JN-3 composite drag reducer is superior to that of the commonly used PPD EVA, and the advantage is more pronounced under high paraffin content conditions. A total of 40 mg/kg is therefore recommended as the effective concentration of the composite drag reducer, beyond which the improvement in the wax inhibition effect is not obvious. From the perspective of application, the composite drag reducer is more suitable for the inhibition of paraffin deposition in pipelines transporting medium- and high-paraffin crude oils, as it achieves a high reduction rate of the paraffin deposition amount at a relatively low concentration and thus offers good application potential.

Additionally, based on the 40 mg/kg as the effective concentration of the composite drag reducer, pour point tests on the model oil containing 30 wt% paraffin without any additive and with PAO/IL/JN-3 composite drag reducer were processed. The pour point depression ΔPP reached 8 °C for the model oil with PAO/IL/JN-3 composite drag reducer. The result confirmed that the composite system also possesses a pour-point-depressing functionality.

#### 2.4.3. Analysis of the Wax Crystal Morphology

As shown in Figure 12, in the model oil containing 30 wt% paraffin without the composite drag reducer (Figure 12A), the paraffin crystals are mainly needle-like, with a large crystal size and a high aspect ratio, interlacing and interlocking with one another to form a dense three-dimensional network; such large-sized, highly oriented paraffin crystal aggregates are the main cause of the low-temperature gelation and the poor fluidity of the model oil.

After the addition of 20 mg/kg of the composite drag reducer (Figure 12B), the paraffin crystal morphology changes obviously: the crystal size decreases significantly, the crystal number increases markedly, and the proportion of needle-like crystals declines, being replaced mainly by fine granular or short rod-like crystals; the degree of interlocking between the paraffin crystals is reduced, the three-dimensional network begins to loosen, and relatively dispersed paraffin crystal distributions have appeared in some local regions. This indicates that the pour point depressant component in the composite drag reducer has begun to adsorb on the surface of the paraffin crystal nuclei or to participate in the co-crystallization, increasing the number of nucleation sites and inhibiting the anisotropic growth of the paraffin crystals.

When the concentration of the composite drag reducer is further increased to 40 mg/kg (Figure 12C), the paraffin crystals are further refined, with the largest number of crystals distributed as fine and uniform granular or punctate particles. Almost no obvious needle-like crystals or large-sized aggregates can be observed; the dispersion of the paraffin crystals is significantly improved, the interlocking phenomenon basically disappears, and the original three-dimensional network structure is effectively destroyed. This indicates that at 40 mg/kg, the composite drag reducer has fully acted on the paraffin crystallization process, fundamentally changing the growth habit and the aggregation mode of the paraffin crystals.

Overall, the regulation of the paraffin crystal morphology by the 40 mg/kg composite drag reducer is evident. The refinement and dispersion of the paraffin crystals can not only reduce the elastic and viscous moduli of the system, but also reduce the adhesion and deposition of the paraffin crystals on the pipe wall or the cold finger surface, thereby synergistically improving the low-temperature fluidity and the paraffin deposition resistance of the high-paraffin crude oil. This is mutually corroborated by the rheological and cold finger experimental results, revealing the microscopic mechanism by which the composite drag reducer weakens the wax crystal network and inhibits wax deposition through the alteration of the paraffin crystal morphology; such refinement and dispersion of wax crystals is generally associated with a weakened gel structure and thus the improved low-temperature flowability of waxy oils [37].

### 2.5. Evaluation of the Drag Reduction Performance

#### 2.5.1. Drag Reduction Rate in the Flow Loop Tests

As shown in Figure 13, the drag reduction rate of the composite drag reducer is significantly affected by both the concentration and the temperature: within the range of 30–60 °C, the drag reduction rate increases continuously with increasing composite drag reducer concentration; at the same concentration, the lower the temperature, the higher the drag reduction rate, although the difference is not obvious.

The drag reduction performance is significant at 30 °C: the drag reduction rate increases with increasing concentration, reaching 50.5% and 54.5% at 40 and 50 mg/kg, respectively. This indicates that at ambient temperature, the molecular chains of the composite drag reducer extend easily and act effectively on the turbulent boundary layer, so that a high drag reduction rate can be obtained with a small concentration. As the temperature increases, the flexibility of the molecular chains of the composite drag reducer increases while their orientation capability weakens, leading to a decline in the drag reduction rate; nevertheless, the PAO polymer chains can still maintain a good extended state and orientation capability. When the temperature rises above 50 °C, the oil viscosity decreases significantly while the thermal motion of the polymer chains intensifies, resulting in a reduced drag reduction rate, indicating that the adverse effect of the intensified thermal motion of the polymer chains must be overcome at high temperature. Each data point in the figure is the average of three independent replicate experiments, with relative standard deviations (RSDs) all below 5%, indicating the good reproducibility of the drag reduction rate test results. These results suggest that for the pipeline transportation of high-paraffin crude oil, the composite drag reducer can be injected directly into the low-temperature pipe sections to obtain a high drag reduction rate.

#### 2.5.2. Drag Reduction Rate in the Field Tests

As shown in Figure 14, during the 0–6 h baseline phase prior to the injection of the composite drag reducer, the system pressure drop (ΔP) remained stable at a relatively high level, with an average value of approximately 2.15 MPa. This reflects the substantial frictional resistance along the high-paraffin crude oil pipeline in the absence of a DRA, which is primarily attributed to the combined effects of viscous dissipation near the pipe wall and the interactions among paraffin crystals. Following the initiation of DRA injection at the position marked by the dashed line at 6 h, the pressure drop decreased rapidly within the 6–12 h interval, declining from the baseline value of approximately 2.15 MPa to approximately 1.15 MPa before reaching a plateau, with the lowest instantaneous pressure drop of 1.02 MPa. This indicates that the PAO/IL/JN-3 composite drag reducer can significantly attenuate the near-wall turbulent fluctuation intensity, thereby achieving rapid drag reduction. Notably, the time elapsed from the onset of injection to the establishment of a stable low-pressure drop was relatively short (approximately 6–8 h), demonstrating that the composite drag reducer exhibits a rapid response and effective dispersion in high-paraffin crude oil.

After 24 h of injection, the system pressure drop stabilized at a low level, with an average value of approximately 1.15 MPa during the 7–30 h period. Compared with the baseline (2.15 MPa), the average drag reduction rate reached approximately 46.5%, demonstrating a remarkable drag reduction performance. The field value is slightly lower than that of the flow-loop test at the same concentration (50.5%), which is mainly attributed to the different media and operating conditions: the flow-loop test used a 20 wt% paraffin model oil under well-controlled conditions, whereas the field pipeline transporting crude oil containing complex composition and shear history can affect the dispersion and effective conformation of the composite drag reducer. This indicates that the composite drag reducer can sustain a stable drag reduction performance under continuous injection, and no significant performance degradation attributable to shear degradation or paraffin crystal deposition was observed within 24 h of continuous operation.

Following the cessation of injection, the system pressure drop exhibited a gradually increasing trend, rising slowly from approximately 1.05 MPa to approximately 1.40 MPa within 5–8 h, yet remaining below the baseline level, which indicates that the residual DRA adsorption layer on the pipe wall retains a certain persistence. This phenomenon suggests that, after the cessation of injection, the DRA molecules within the pipeline are gradually washed out and the PAO adsorption layer on the pipe wall progressively thins, leading to the gradual recovery of frictional resistance. This further demonstrates that continuous injection of the composite drag reducer is required to maintain a stable drag reduction performance. In summary, the composite drag reducer exhibits the characteristics of rapid response, substantial drag reduction, and favorable stability in high-paraffin crude oil pipeline transportation, making it suitable for energy conservation and consumption reduction in continuously transported high-paraffin crude oil pipelines. It should be emphasized that the mechanistic conclusions on wax crystal regulation were established in model oil systems containing 20–30 wt% paraffin, and their extrapolation to real crude oils with more complex compositions should be made with caution.

## 3. Materials and Methods

### 3.1. Materials

Poly(α-olefin) suspension (PAO, milky white viscous liquid, active content 30 wt%, MW. 8–12 × 10^6^ g/mol) and the pour point depressant JN-3 (transparent dark yellow liquid, active content 42 wt%, MW. ~2 × 10^4^ g/mol) were purchased from Jiahua Technology Co., Ltd. (Jingzhou, China). The model oils were prepared from refined paraffin and No. 0 diesel (purchased from a local gas station) at different mass ratios. Paraffin (solid), ethylene-vinyl acetate copolymer (EVA), 1-dodecyl-3-methylimidazolium bromide (IL-C12, 97%), 1-tetradecyl-3-methylimidazolium bromide (IL-C14, 97%), 1-hexadecyl-3-methylimidazolium bromide (IL-C16, 97%), and 1-benzyl-3-methylimidazolium bromide (IL-Ben, 97%) were purchased from Aladdin Reagent Co., Ltd. (Shanghai, China). All materials were used as received without further purification.

### 3.2. Preparation and Proportion Optimization of the Composite Drag Reducer

The PAO/IL/JN-3 ternary composite drag reducer was prepared by a co-dispersion method. PAO was dispersed into an oil-based suspension system at different mass ratios to obtain the PAO stock solution; the JN-3 pour point depressant and the ionic liquid were then added into the PAO stock solution at different ratios and mixed by stirring until uniform, and the product was denoted as PAO/IL/JN-3. The mixture was stirred slowly at room temperature for 120 min to ensure uniform dispersion. Several control groups, such as the PAO/EVA and PAO/IL/EVA blends, were also prepared to verify the effectiveness of the PAO/IL/JN-3 ternary composite drag reducer.

### 3.3. Characterization Methods for the Composite Drag Reducer

The FT-IR spectrum of the PAO/IL/JN-3 composite drag reducer was recorded on a Nicolet 6700 (Thermo Fisher, Waltham, MA, USA) spectrometer over 4000–400 cm^−1^ at a scan rate of 20 scans/min. The crystallization/melting behavior of the PAO/IL/JN-3 composite drag reducer was measured using a differential scanning calorimeter (TA DSC 250, TA Instruments, New Castle, DE, USA). Under a nitrogen atmosphere, the sample was cooled from 80 °C to −40 °C at a cooling rate of 10 °C/min, and then heated back to 80 °C at 10 °C/min, to analyze the physical interactions among the three components.

The thermal decomposition curve of the PAO/IL/JN-3 composite drag reducer was measured using a thermogravimetric analyzer (TG 209 F3, NETZSCH, Selb, Germany) under a nitrogen atmosphere, heating from room temperature to 800 °C at a heating rate of 10 °C/min, in order to evaluate the thermal stability of the composite drag reducer.

### 3.4. Performance Test Methods

The following performance test methods were used:

(1) Rheological test. The rheological properties of the samples during the thermal conditioning process were measured using a HAAKE MARS 60 rheometer (Thermo Fisher Scientific, Darmstadt, Germany). A model oil containing 20 wt% paraffin was prepared by adding paraffin at 20 wt% into the diesel; the composite drag reducer was added into the oil samples at 0–50 mg/kg, followed by stirring at a constant temperature of 60 °C for 20 min to ensure the uniform dispersion of the additive. The storage modulus (G’), loss modulus (G″), and torque (M) were then continuously recorded as functions of temperature and time using a coaxial cylinder geometry, the temperature was decreased from 40 °C to 20 °C at 1 °C/min, and the oscillatory measurements were performed at a fixed frequency of 1Hz within the linear viscoelastic region.
(2) Paraffin deposition test. The effect of the composite drag reducer on the paraffin deposition behavior of the model oils was tested by the cold finger method. A model oil containing 30 wt% paraffin was prepared by adding paraffin at 30 wt% into the diesel, the composite drag reducer was added into the oil samples at 0–50 mg/kg and stirred at 45 °C to ensure the uniform dispersion of the additive; during the deposition test, the model oil was maintained at 45 °C and kept string, following the reported cold finger methodology [38]. The effects of the blend control groups at the same concentration were also compared. The cold finger was a stainless-steel tube with an outer diameter of 25 mm and an effective immersion depth of 60 mm; a circulating coolant controlled precisely at 5 °C by a low-temperature thermostat bath was passed through the cold finger. The cold finger was immersed into the model oil and taken out at different time points within 1–10 h; the paraffin deposited on its surface was weighed, and the paraffin deposition amount at each time point was recorded.
(3) Pour point test. The pour point of each sample was determined according to GB/T 3535 [32]. A model oil containing 30 wt% paraffin was heated in a water bath at 45 °C to completely dissolve the paraffin. The specimen was poured into the standard test jar to the level mark, and a cork-mounted thermometer was positioned with the beginning of its capillary 3 mm below the liquid surface. The jar was placed in a jacket and transferred successively through a cooling bath at 0 °C. Starting from about 9 °C above the expected pour point, the jar was withdrawn at every 3 °C interval, held in the horizontal position for 5 s, and examined for movement of the liquid surface. The pour point was taken as 3 °C above the temperature at which no movement was observed. Each sample was analysed at least three times. The extent of pour point depression was quantified as ΔPP = PP_0_ − PP, where PP_0_ and PP denote the pour points of model oil without additive and with composite drag reducer, respectively.
(4) Paraffin crystal morphology observation. A polarizing optical microscope equipped with a temperature-controlled cold stage was used to observe the changes in the morphology and distribution of the paraffin crystals in the 30 wt% paraffin model oil before and after the addition of the additive, and to analyze the intervention effect of the composite drag reducer on the paraffin crystal growth habit.
(5) Drag reduction rate flow loop test and field test. A circulating flow loop apparatus (tube inner diameter 10 mm, tube length about 19 m) was employed. At flow rate 0.8 m^3^/h under 30 °C–60 °C temperature, the pressure drop Δ*P*_0_ of the 20 wt% paraffin model oil passing through the test section was recorded; subsequently, the pressure drop Δ*P_DR_* after the addition of a certain concentration (5–50 mg/kg) of the composite drag reducer under the same temperature and flow rate conditions was recorded, and the drag reduction rate *DR* was calculated using Equation (1). Each measurement was repeated at least three times. The relevant equation is defined as follows:

(1) $$\mathrm{DR}=\frac{{∆P}_{0}-{∆P}_{\mathrm{DR}}}{{∆P}_{0}}\times 100\%$$

The field test was conducted on an outdoor oil transportation pipeline at a domestic gathering station (D150 mm × 34 km) with a crude oil throughput of 570 t/h. The crude oil in the pipeline had a density of 820 kg/m^3^, a viscosity of 6.7 mPa·s, a pour point of 18 °C, a wax content of 24.1 wt%, a wax appearance point of 27 °C, a resin content of 0.95%, and an asphaltene content of 1.15%; the carbon number distribution was as follows: <C_16_ 67.30%, C_16_–C_25_ 24.78%, C_26_–C_30_ 5.93%, and >C_31_ 1.99%. During the test, the composite drag reducer was continuously injected into the oil transportation pipeline through the injection point at 40 mg/kg for 30 h using a diaphragm metering pump, the pressure drops Δ*P*_0_ and Δ*P_DR_* of the pipeline were recorded, and the drag reduction rate *DR* was calculated using Equation (1).

## 4. Conclusions

In this study, a PAO/IL/JN-3 ternary composite drag reducer was constructed via physical blending, using PAO as the drag-reducing main agent, JN-3 as the pour point depressant component, and an ionic liquid as the functional regulator, and its application performance was systematically evaluated. The proportion optimization results showed that the optimal mass ratio of PAO to JN-3 was 1:2, IL-C14 provided the best enhancement, and the optimal mass fraction of IL was 30 wt%. The ionic liquid interacts with the polar functional groups of JN-3 through its polar imidazolium group and undergoes hydrophobic association with the PAO backbone and the paraffin crystals through its long alkyl chain, and it is hypothesized to form a “molecular bridge” between the two functional components, which may account for the observed synergistic effect and requires further direct verification. The FT-IR, DSC, and TGA characterization results showed that the characteristic peaks of the three components coexisted independently without any new peaks; no glass transition, crystallization, or melting event was observed within the pipeline-relevant range of −40 °C to 80 °C; and the weight loss of the composite drag reducer below 340 °C was only 7.15%, indicating good compatibility and a thermal stability that satisfies the pipeline transportation requirements. The rheological tests showed that at a concentration of 40 mg/kg, the storage modulus G’ at 30 °C was approximately 82% lower and the loss modulus G″ was approximately 78% lower than those of the PAO-only system, and the torque decreased by 65–80%, indicating a significant improvement in the low-temperature rheological properties. The paraffin deposition evaluation showed that for the model oil containing 30 wt% paraffin, the reduction rate of the paraffin deposition amount reached 38% at 40 mg/kg, the deposition amount at 0.5 h was 17.6% lower than that of the blank group at 40 mg/kg, and the rapid growth stage of paraffin deposition was obviously delayed; the paraffin crystals transformed from coarse needle-like to fine granular, and the three-dimensional network structure was effectively destroyed. The drag reduction performance evaluation showed that in the flow loop tests at 30 °C, the drag reduction rates reached 50.5% and 54.5% at 40 and 50 mg/kg, respectively; the drag reduction rate decreased slightly with increasing temperature, but a good effect could still be maintained at concentrations above 40 mg/kg. In the field pipeline test, after the continuous injection of 40 mg/kg, the pipeline pressure decreased from 2.15 MPa to 1.15 MPa, with an average reduction of 46.5%, together with a fast response and good stability. Overall, the PAO/IL/JN-3 ternary composite drag reducer combines drag reduction and pour point depression in a synergistic manner, with 40 mg/kg identified as the recommended effective concentration.

Although the ternary composite drag reducer developed in this study provides a theoretical basis for the transportation of real crude oils, the specific field conditions should still be considered in practical applications. Furthermore, issues such as the relatively high cost of the ionic liquid, its potential environmental impact, and its recyclability remain to be systematically investigated.

## Figures and Tables

**Figure 1:**
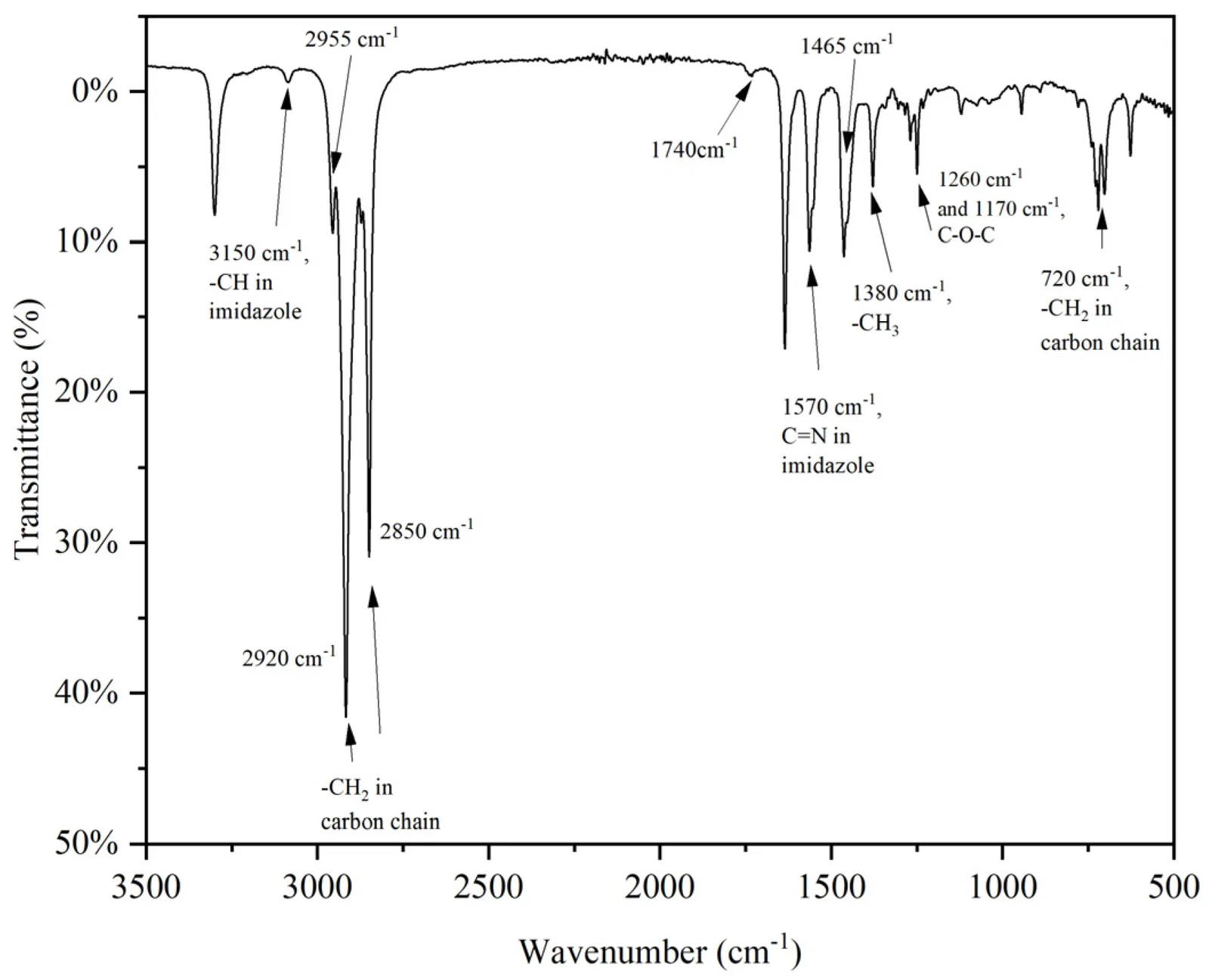
FT-IR spectrum of the PAO/IL/JN-3 composite drag reducer.

**Figure 2:**
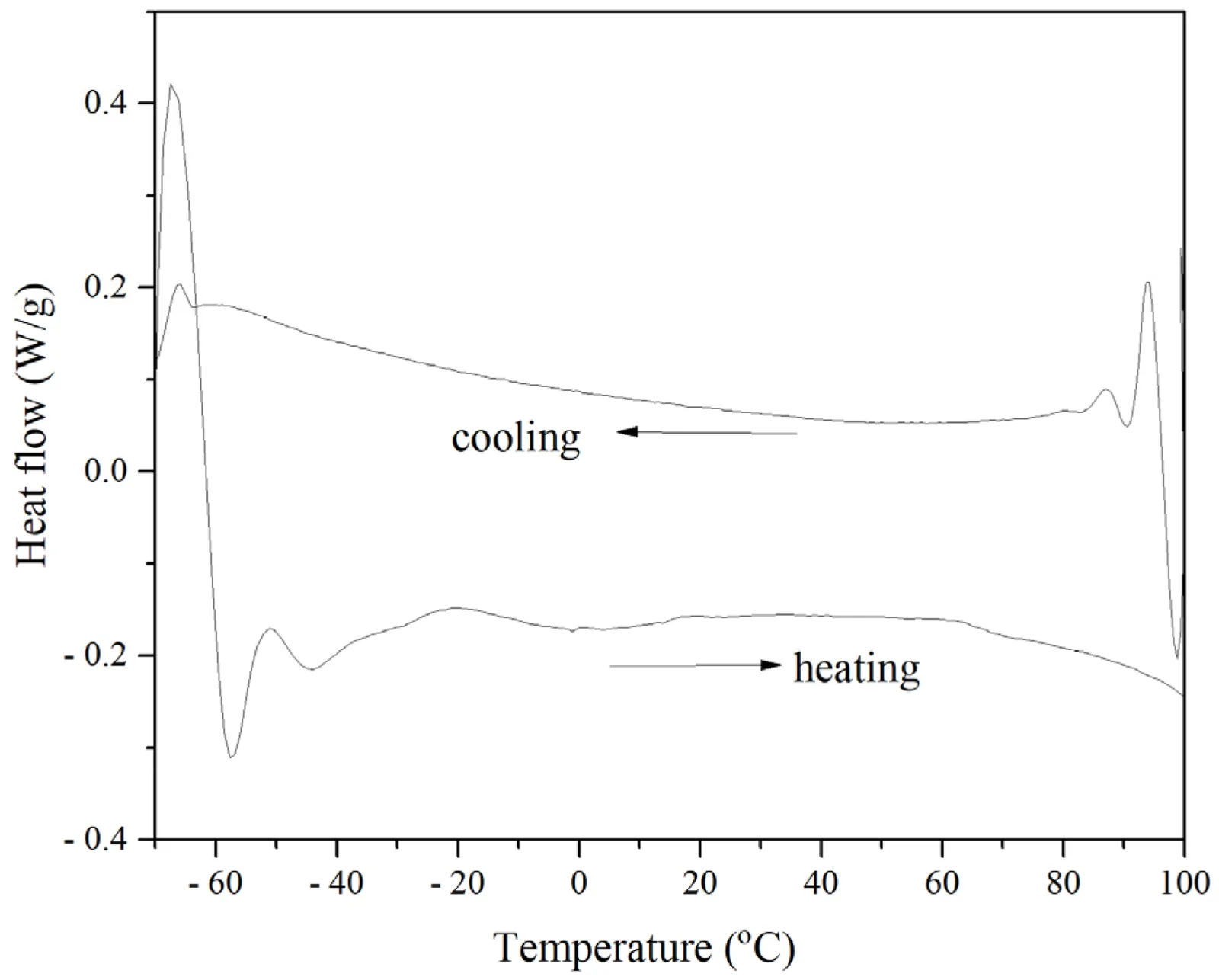
DSC curves of the PAO/IL/JN-3 composite drag reducer.

**Figure 3:**
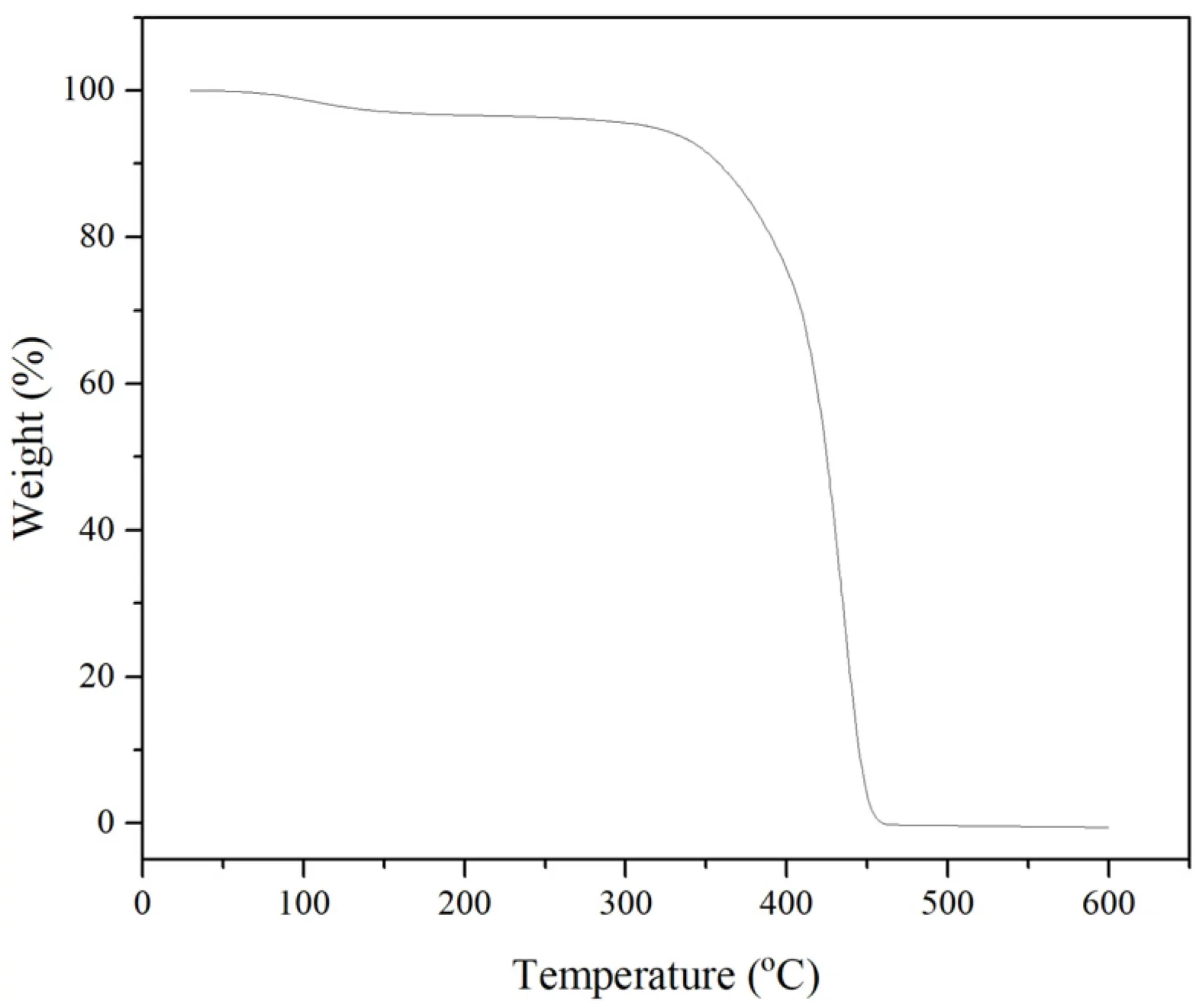
TGA curves of the PAO/IL/JN-3 composite drag reducer.

**Figure 4:**
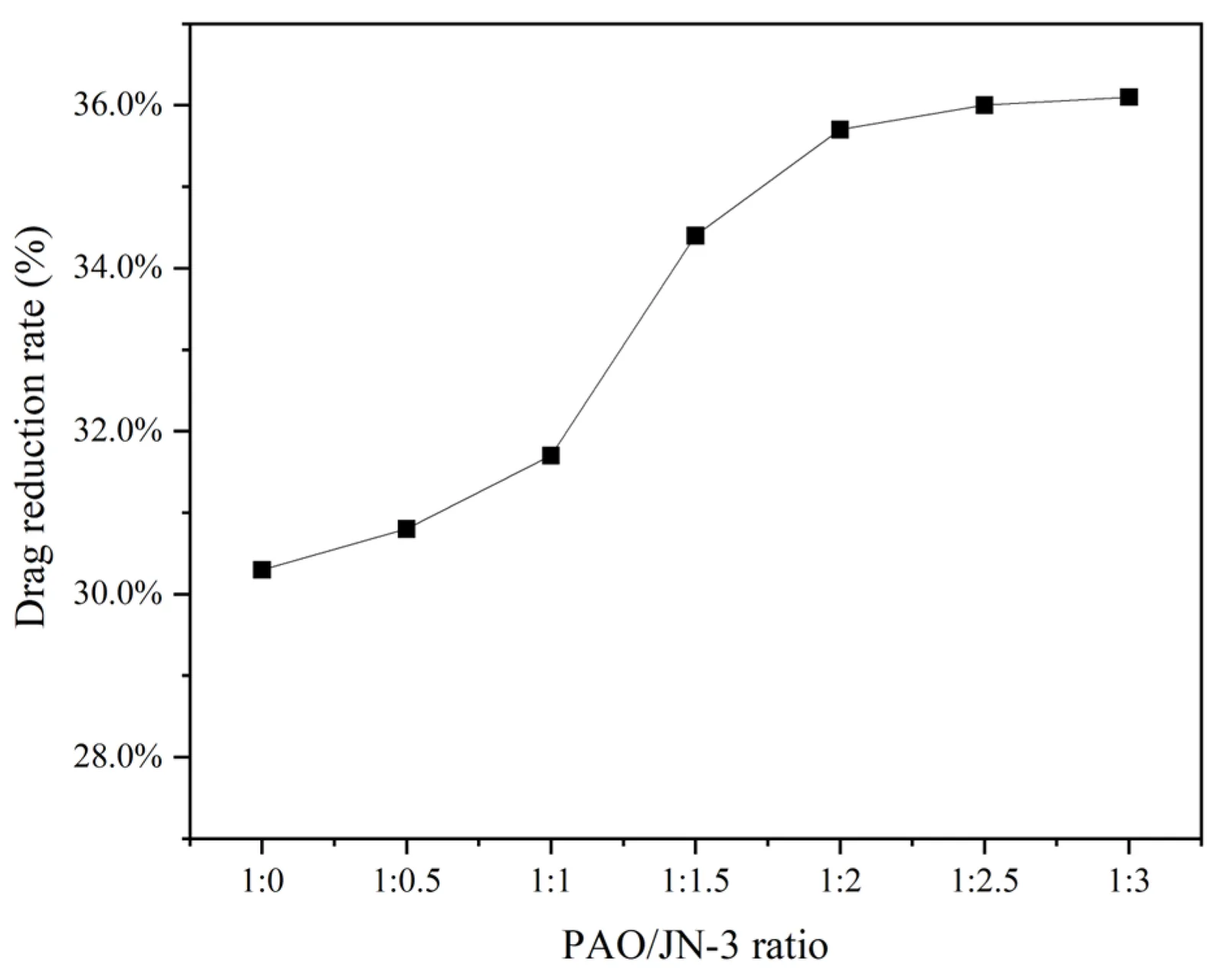
Effect of the PAO/JN-3 mass ratio on the drag reduction rate.

**Figure 5:**
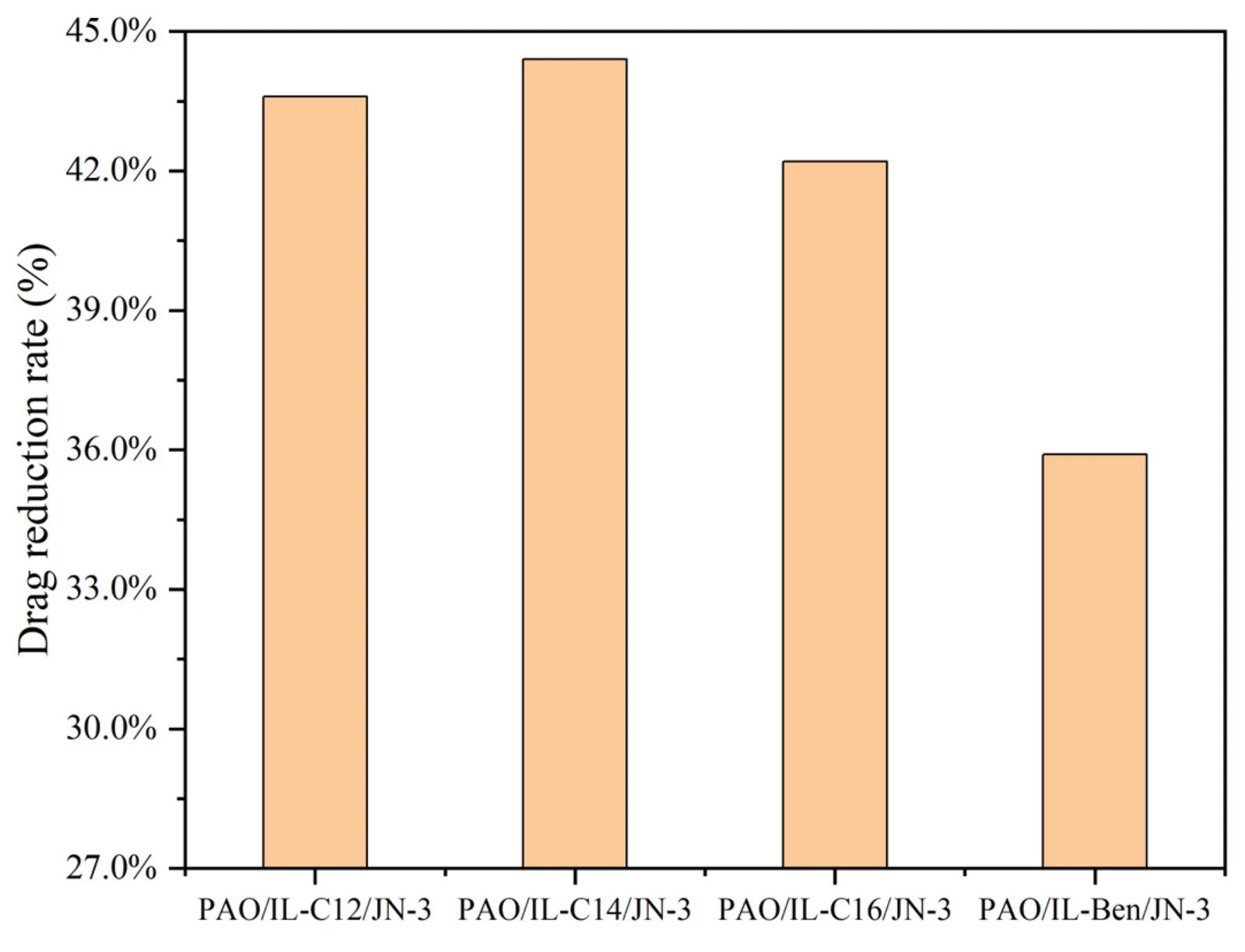
Effect of the molecular structure of different ionic liquids on the drag reduction rate.

**Figure 6:**
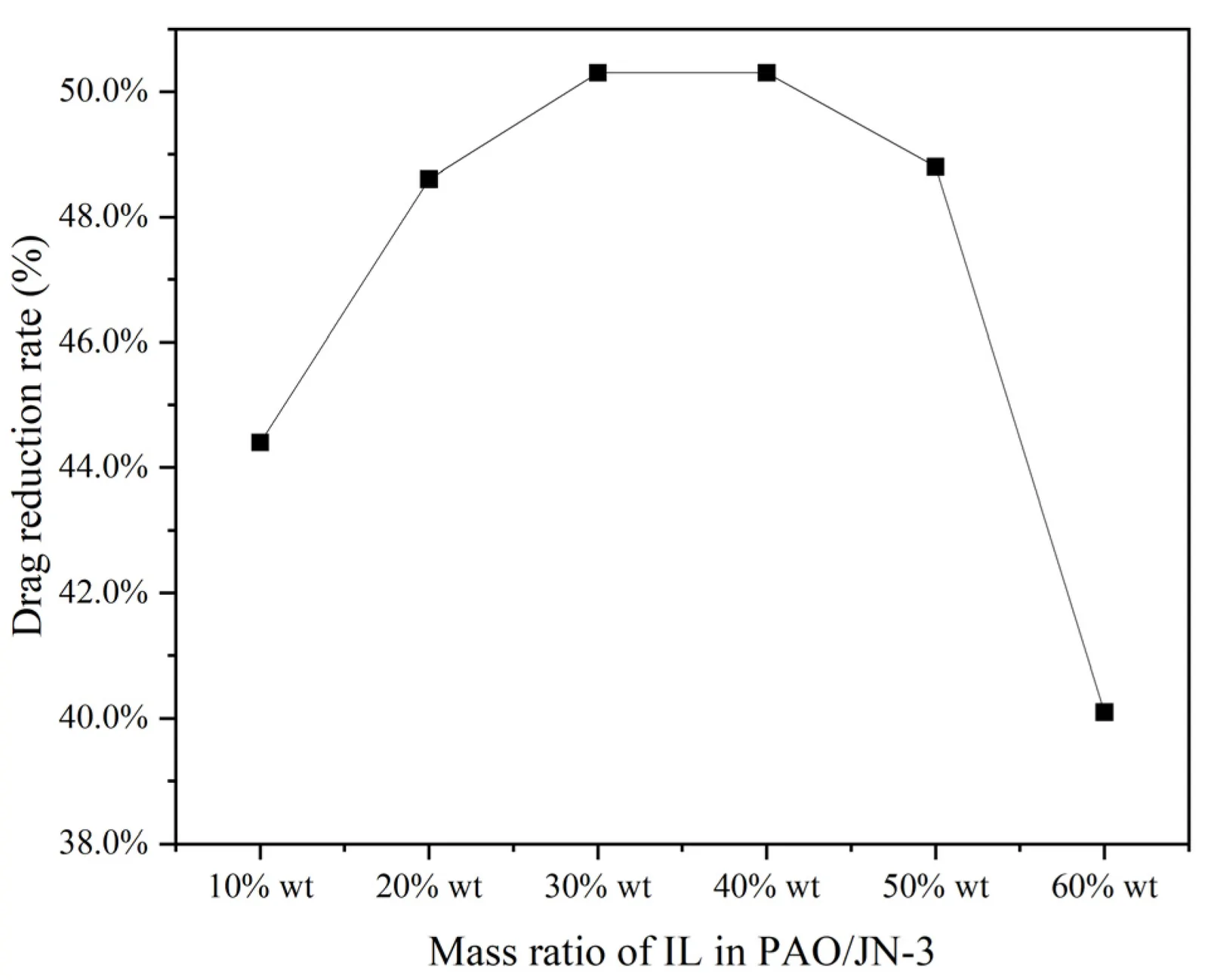
Effect of the mass fraction of the ionic liquid (IL-C14) in the PAO/IL/JN-3 ternary composite drag reducer on the drag reduction rate.

**Figure 7:**
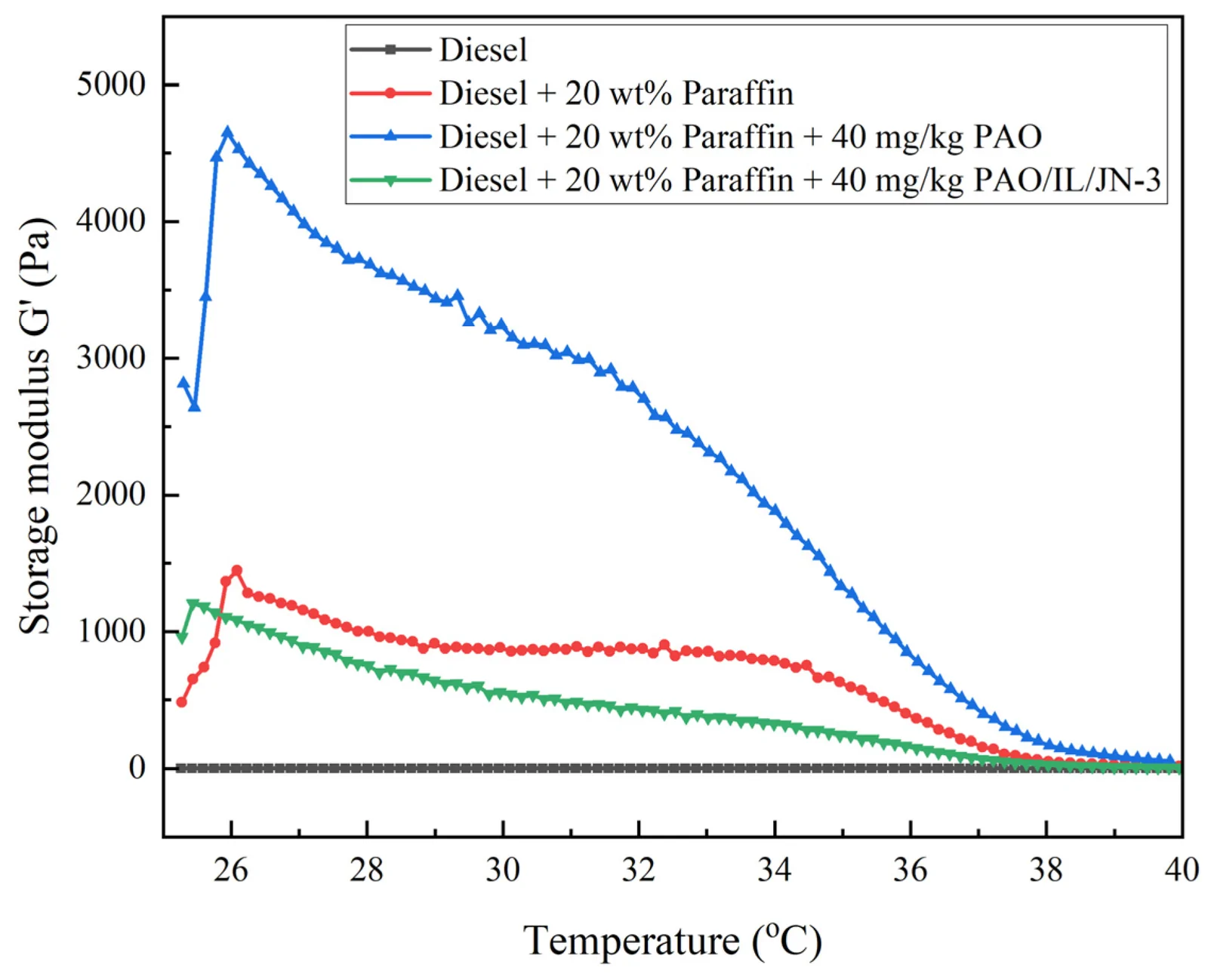
Storage modulus G’ of the model oil before and after the addition of the PAO/IL/JN-3 composite drag reducer.

**Figure 8:**
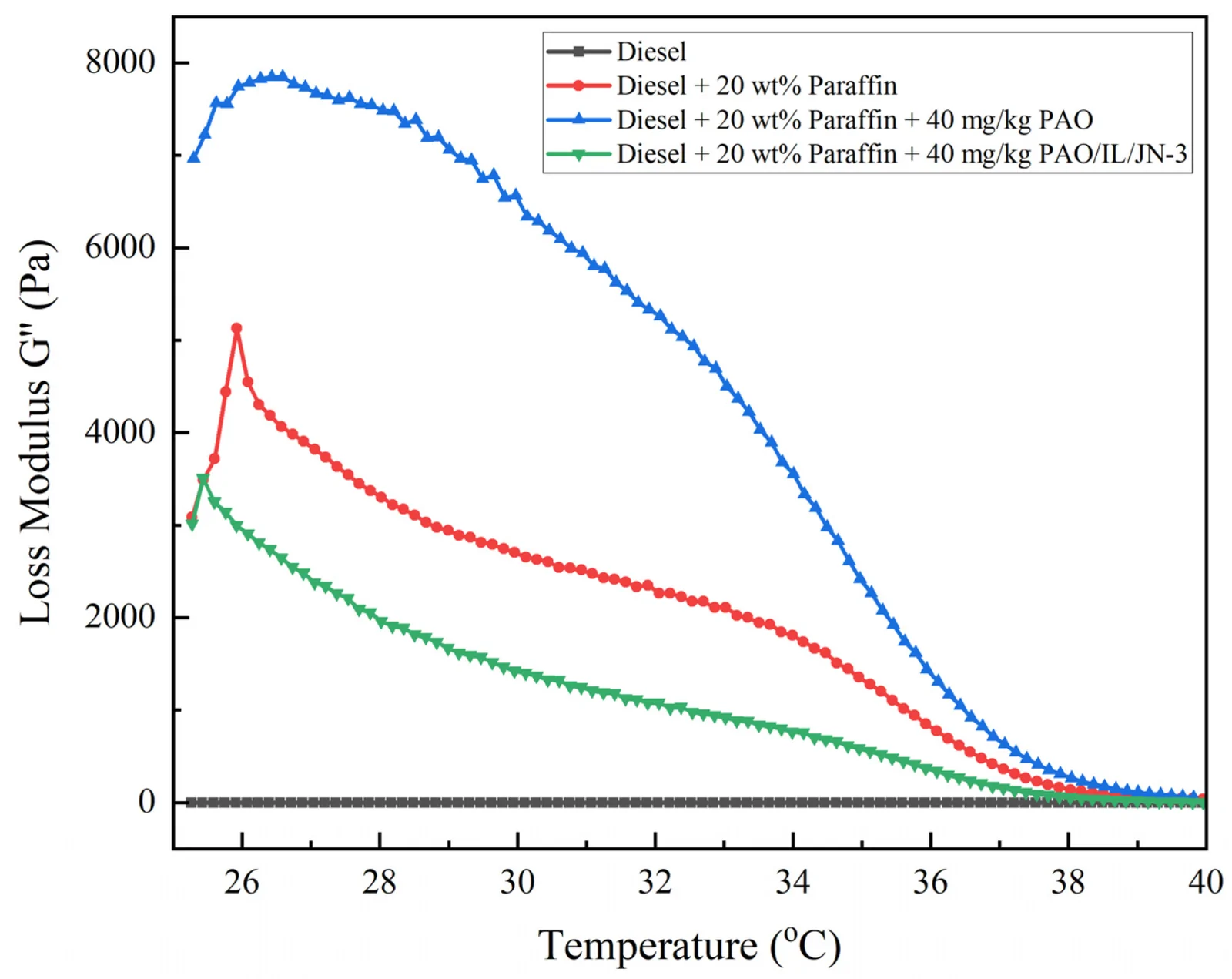
Loss modulus G″ of the model oil before and after the addition of the PAO/IL/JN-3 composite drag reducer.

**Figure 9:**
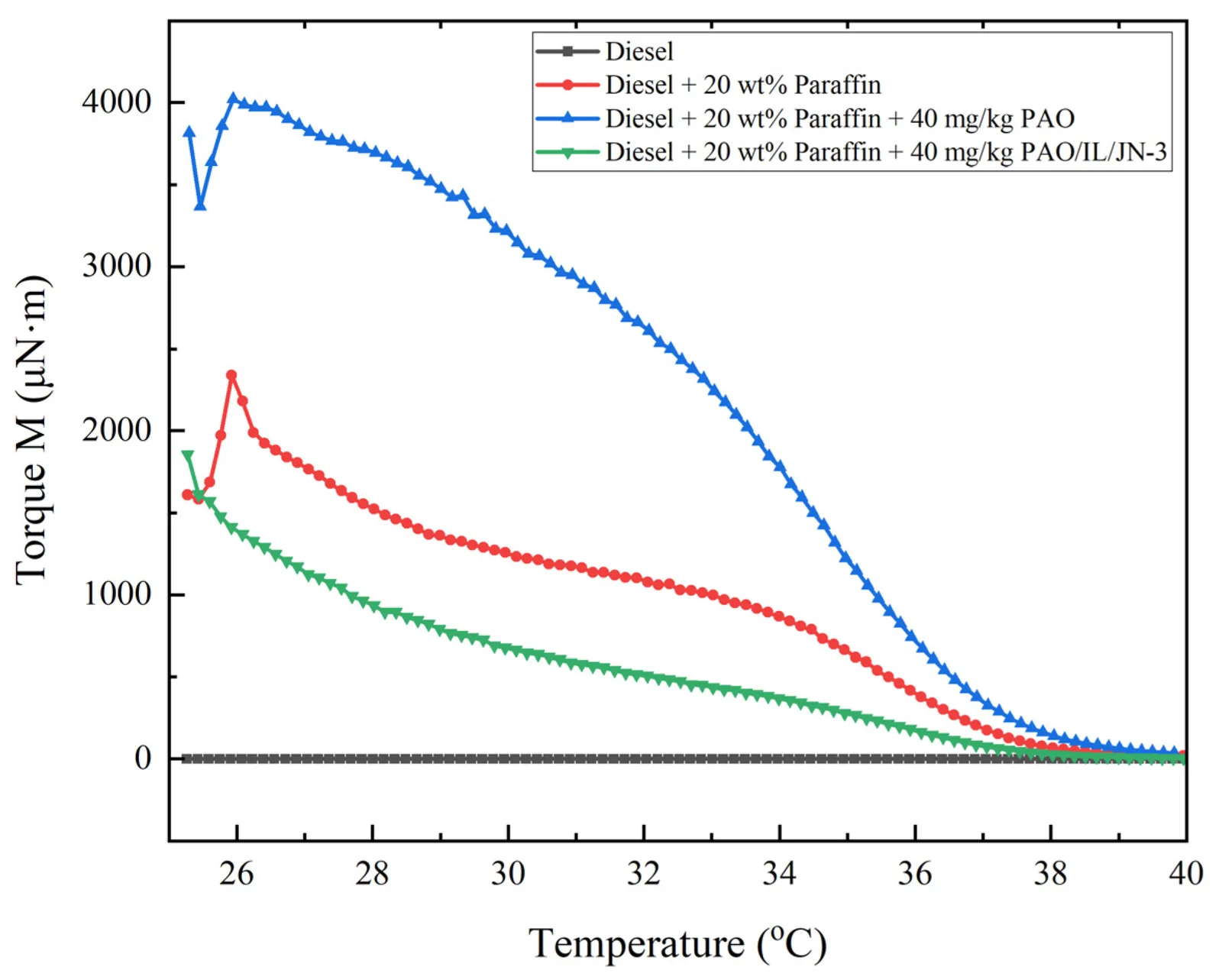
Torque M of the model oil before and after the addition of the PAO/IL/JN-3 composite drag reducer.

**Figure 10:**
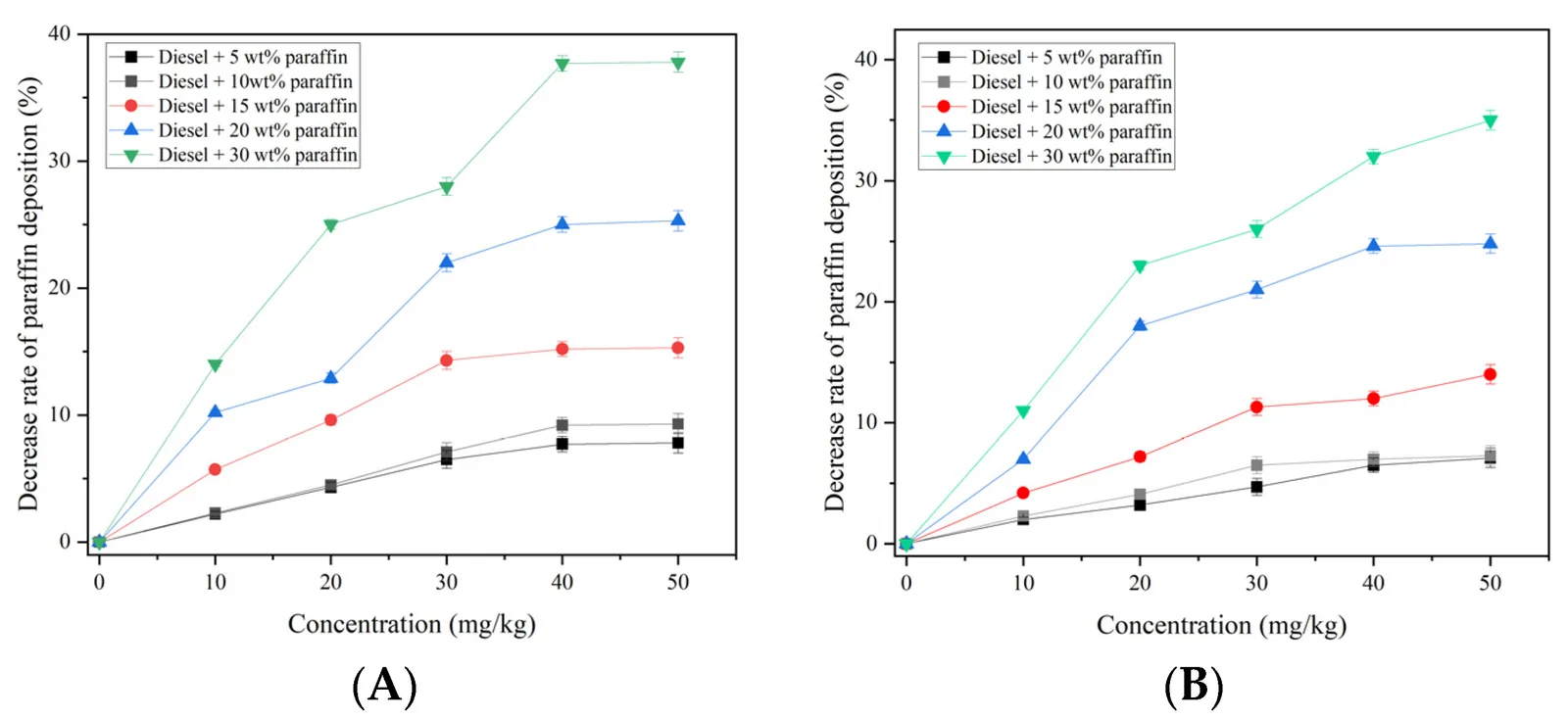
Effect of the composite drag reducers on the reduction rate of the paraffin deposition amount of the model oils under identical conditions: (**A**) PAO/IL/JN-3 composite drag reducer; (**B**) PAO/IL/EVA composite drag reducer. Error bars represent the standard deviations of three independent replicates.

**Figure 11:**
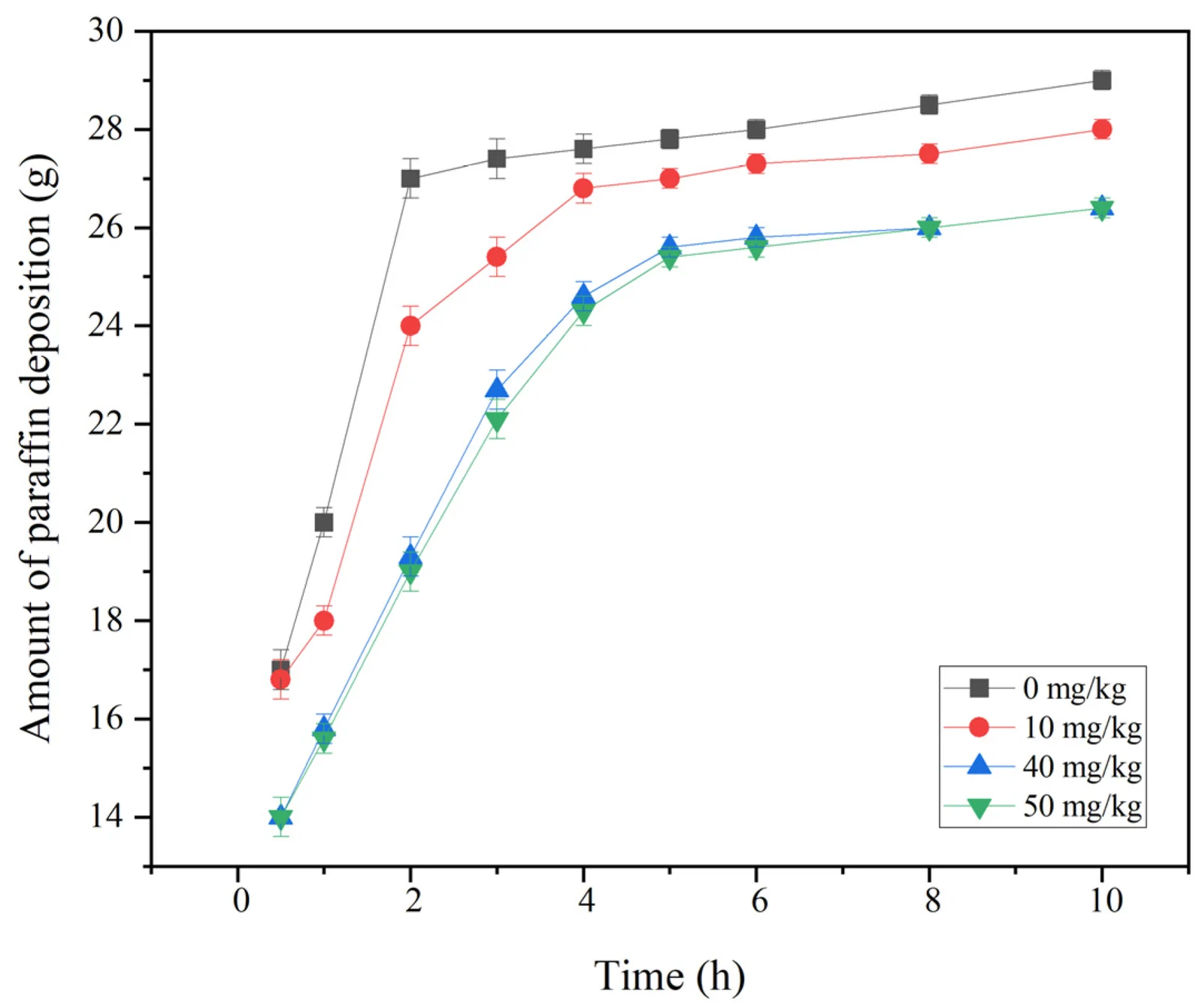
Effect of the PAO/IL/JN-3 composite drag reducer on the paraffin deposition behavior of the model oil containing 30 wt% paraffin. Error bars represent the standard deviations of three independent replicates.

**Figure 12:**
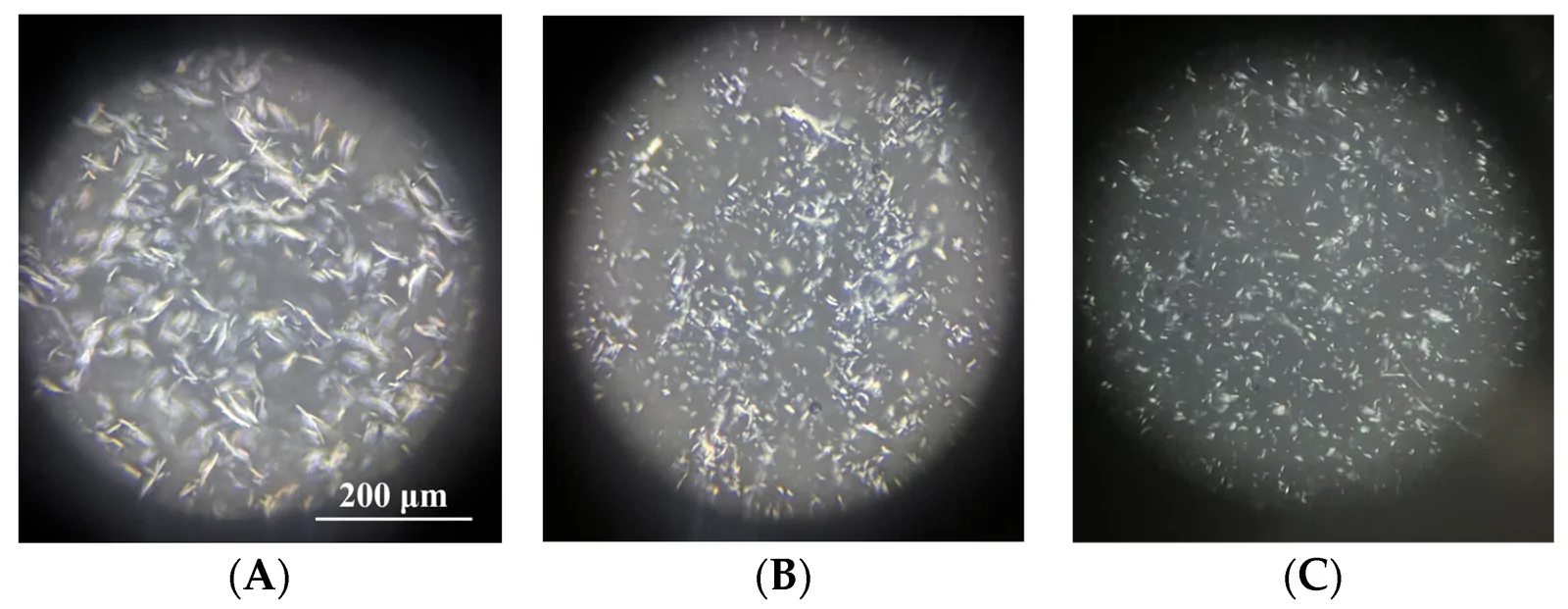
Paraffin crystal morphology under the microscope: (**A**) without additive, (**B**) with 20 mg/kg PAO/IL/JN-3 composite drag reducer, and (**C**) with 40 mg/kg PAO/IL/JN-3 composite drag reducer.

**Figure 13:**
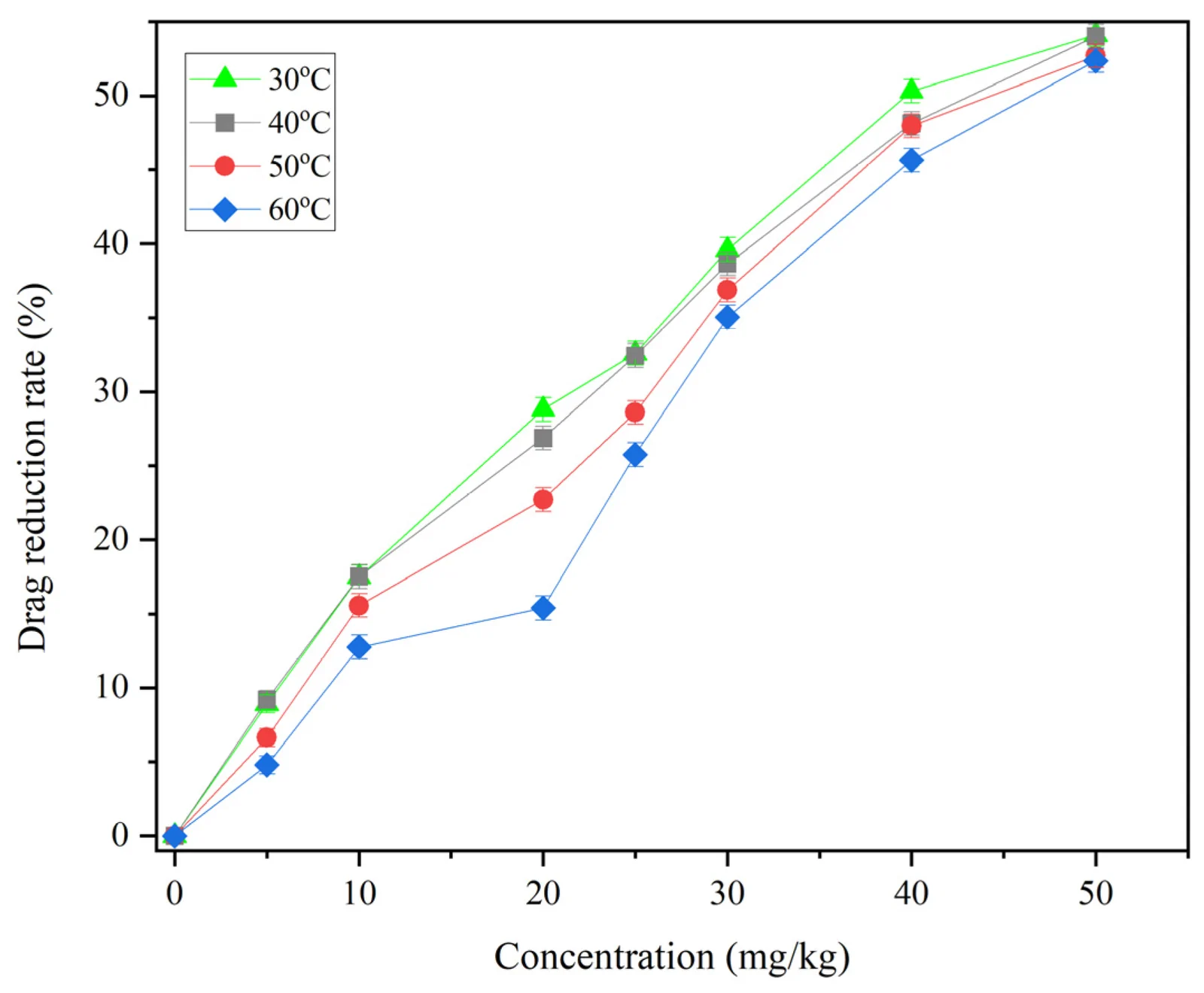
Effect of the PAO/IL/JN-3 composite drag reducer concentration on the drag reduction rate in the flow loop tests.

**Figure 14:**
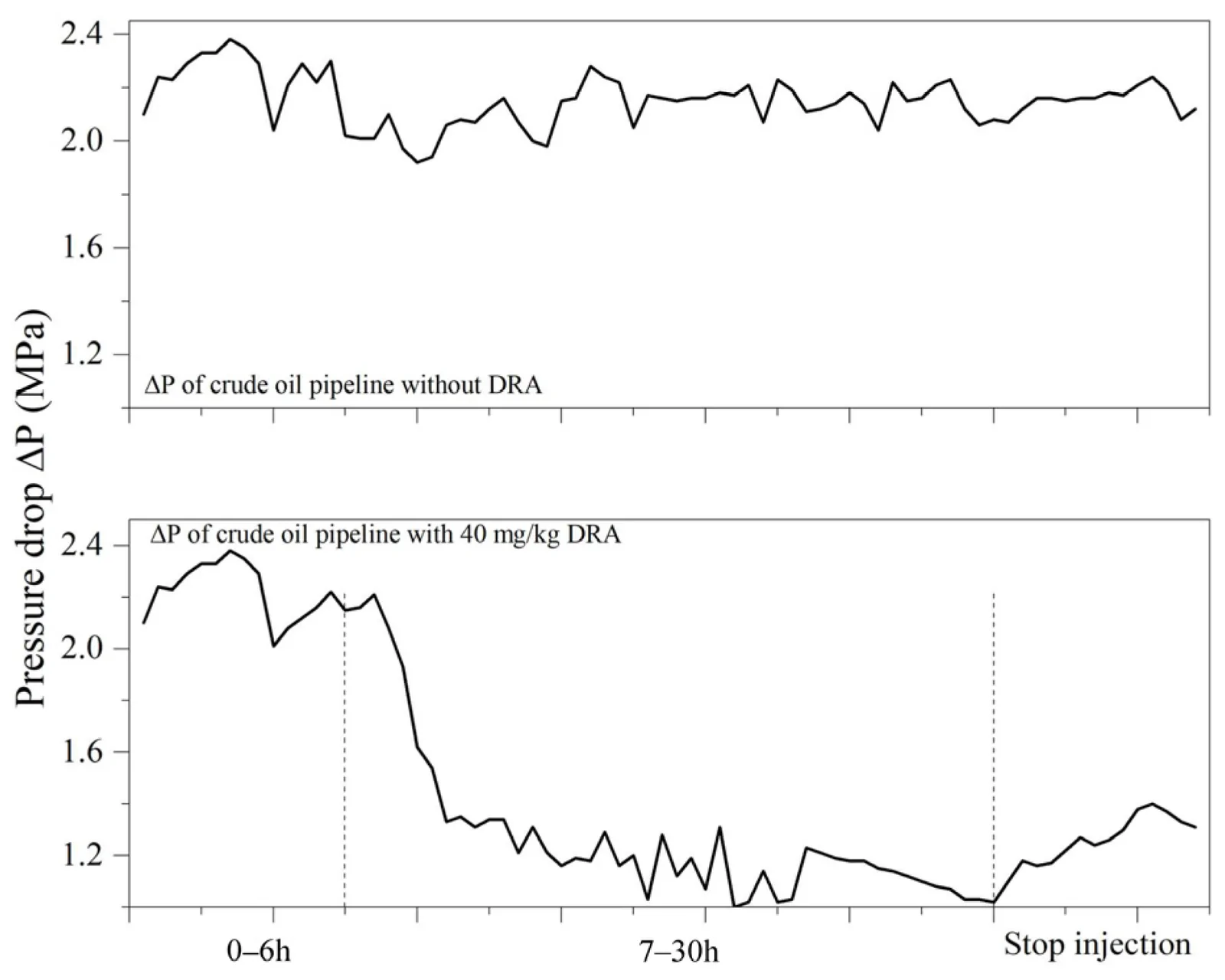
Trend of the pressure drop of the oil transportation pipeline in the field test.

## Data Availability

Data are contained within the article.
